# Detection and management of localized prostate cancer in Nigeria: barriers and facilitators according to patients, caregivers and healthcare providers

**DOI:** 10.1186/s12913-024-11340-1

**Published:** 2024-08-12

**Authors:** Musliu Adetola Tolani, Christian A. Agbo, Alan Paciorek, Shehu S. Umar, Rufus W. Ojewola, Faruk Mohammed, Ernie Kaninjing, Muhammed Ahmed, Rebecca DeBoer

**Affiliations:** 1 Ahmadu Bello University / Ahmadu Bello University Teaching Hospital, P.M.B. 06, Shika- Zaria, Kaduna State Nigeria; 2 Dalhatu Araf Specialist Hospital, Shendam Road, Lafia, Nasarawa State Nigeria; 3 University of California, San Francisco, Box 0874, San Francisco, CA 94110 USA; 4 University of Lagos, Lagos University Teaching Hospital, Idi-Araba, Lagos State Nigeria; 5 Georgia College and State University, Campus Box 112, Milledgeville, GA 31061 USA; 6 Division of Urology, Department of Surgery, Ahmadu Bello University Teaching Hospital, P.M.B. 06, Shika-Zaria, Kaduna State Nigeria

**Keywords:** Delivery of health care, Health services accessibility, Needs assessment, Nigeria, Prostatic neoplasms, Qualitative research

## Abstract

**Background:**

Prostate cancer mortality rates are high in Nigeria. While prostate cancer is highly curable with early detection and effective multidisciplinary management, the quality of care is suboptimal in this setting. Sustainable delivery of high-quality care for patients with localized prostate cancer is needed to save more lives. To inform future interventions to improve care, this study aimed to identify barriers and facilitators that influence prostate cancer detection and management in Nigeria.

**Methods:**

Six focus group discussions (FGDs), stratified by stakeholders were conducted with a purposive sample of prostate cancer patients (*n* = 19), caregivers (*n* = 15), and healthcare providers (*n* = 18), in two academic tertiary hospitals in northern and southern Nigeria. A discussion guide organized based on the socio-ecological model was used. FGDs were recorded, transcribed, and analysed using the framework technique.

**Results:**

Barriers and facilitators were identified at the individual, interpersonal, and organizational levels. Barriers to detection included limited knowledge and misperceptions among patients, caregivers, and community-based non-specialist healthcare providers, and limitations of centralized opportunistic screening; while facilitators included the potential for religious institutions to encourage positive health-seeking behaviour. Barriers to management included non-uniformity in clinical guideline usage, treatment abandonment amidst concerns about treatment and survival, absence of patient interaction platforms and follow-up support systems, difficulty in navigating service areas, low health insurance coverage and limited financial resource of patients. Facilitators of management included the availability of resource stratified guidelines for prostate cancer management and the availability of patient peers, caregivers, nurses, and medical social workers to provide correct medical information and support patient-centred services. Participants also provided suggestions that could help improve prostate cancer detection and management in Nigeria.

**Conclusion:**

This study identified multiple determinants affecting the detection and management of localized prostate cancer. These findings will inform the refinement of implementation strategies to improve the quality of prostate cancer care in Nigeria.

## Background

The burden of prostate cancer in Nigeria, a lower middle-income country on the west coast of Africa, is rising. Specifically, the incidence of this cancer increased from 11,944 in 2014 to 15,306 in 2020 [1, 2]. Wide disparities in the mortality-to-incidence ratio in different geographical contexts (i.e., 0.56 in Western Africa versus 0.16 in Northern America and 0.19 in Western Europe) [3], illustrate the glaring inequity in prostate cancer management outcomes between Low and Middle-Income Countries (LMICs) and High-Income Countries (HICs).

Clinically localized prostate cancer means T1─T3a, N0,M0 disease [4]. Curative treatment, which may include active surveillance, radical prostatectomy, radical radiotherapy, and/or androgen deprivation therapy, is recommended for this early stage of cancer. However, the pathways for screening and early diagnosis, staging, treatment, and follow-up in these patients are complex, involving all tiers of the healthcare system and multidisciplinary specialists. A National Cancer Control Plan (2018–2022) was published as a framework to address gaps in priority areas for cancer detection and management in Nigeria [5]. However, the aspects of community-based approaches to detection, guideline use, treatment access, and survivorship care, remain largely unimplemented during the delivery of care to these patients. Reduction in prostate cancer mortality rates will require increased attention to the detection and cure of early-stage disease. Although some researches have been conducted to characterize the barriers and facilitators of common cancers, like breast and cervical cancers, in Africa; there is paucity of research to comprehensively study the determinants of prostate cancer detection and management in this geographical context. The characteristics of prostate cancer patients are quite unique and different from those of these cancers; hence the need to holistically study these determinants.

Interventions are also needed to sustainably strengthen the care delivery system for localized prostate cancer in Nigeria. Needs assessment represents an important phase of intervention development by creating a better understanding of contextual factors that have significant potential to influence the implementation of tailored pilot interventions and scale-up of future programs. To inform the future development of interventions to improve care, this study aimed to identify barriers and facilitators that influence the detection and management of clinically localized prostate cancer in the country.

## Methods

### Setting

Nigeria is the largest country in Africa. The study was carried out at two public tertiary hospitals and designated comprehensive cancer care centres in different geopolitical zones: Ahmadu Bello University Teaching Hospital, a 730-bed hospital in Zaria in the Northwest Zone, and Lagos University Teaching Hospital, a 950-bed hospital in Lagos in the Southwest Zone. These facilities include prostate cancer care as part of the oncology services provided. Diagnostic services include core biopsy of the prostate with histopathology; laboratory services; and computed tomography (CT) and magnetic resonance imaging. Treatment services include radical prostatectomy, external beam radiotherapy with linear accelerator or cobalt-60, brachytherapy, and androgen deprivation therapy with GnRH agonists. Payment for clinical care is predominantly through a fee-for-service model. These centres receive referrals from primary and secondary healthcare centres where the delivery of preventive oncology services is not structured.

### Study design

This qualitative research, with a phenomenological study design, was conducted from August to September 2022. It utilized focus group discussions. Focus groups provided an avenue for researchers to gain an in-depth understanding of participants’ experiences regarding prostate cancer detection and management in Nigeria. Focus groups also provided an opportunity to capture the context in which the health behaviour occurred, and how treatment and cure were provided. These are important topical areas given the dearth of research among this population. Socio-ecological model was used as the theoretical framework for this study as its levels has important determinants and can facilitate the design of a comprehensive intervention that holistically addresses various dimensions of the barriers and facilitators [6].

### Sampling, participant eligibility, and recruitment

Purposive sampling was used to select a diverse group of participants [7]. Eligible patients included those who were diagnosed with clinically localized prostate cancer from all risk groups of prostate cancer and have been managed at the study site for a period of not less than 6 months. Eligible caregivers included family caregivers and informal caregivers such as neighbours and friends above the age of 18 years, and professional caregivers such as medical social workers, who must have been present in a minimum of one prostate cancer-related hospital visit at the site to offer healthcare support to study-eligible patients. Eligible healthcare providers included those with at least 3 years of experience in the management of prostate cancer in the specialties of urology, radiology, pathology, clinical oncology, radiation oncology and oncology nursing. The patients and caregivers were identified during prostate cancer clinic visits while the staff list at the study site was used to identify the healthcare providers. These participants were recruited through in-person or email invitation four weeks before the FGD session, and their written informed consent was obtained.

### Data collection

Study participants were stratified into three groups: **patients**, **caregivers**, and **healthcare providers**. Informed consent was obtained from all participants. A short demographic survey, to capture the profile of study participants, in terms of age, gender, geographic region, educational attainment, and employment status, was completed prior to each focus group. Participant characteristics are summarized on Table 1.

**Table 1:** Demographic detail of the study participants (n = 52)

Characteristics*	Overall	Patients	Caregivers	Providers
**Number of participants**	52	19	15	18
**Age (years)**	56 (30–77)	70 (56–77)	47 (30–75)	40 (30–63)
**Gender**				
Female	11 (21)	0 (0)	8 (53)	3 (17)
Male	41 (79)	19 (100)	7 (47)	15 (83)
**Geographical zone**				
North	26 (50)	10 (53)	6 (40)	10 (56)
South	26 (50)	9 (47)	9 (60)	8 (44)
**Education**				
No education	1 (2)	1 (5)	0 (0)	0 (0)
Secondary education	5 (10)	3 (16)	2 (13)	0 (0)
Tertiary education	46 (88)	15 (79)	13 (87)	18 (100)
**Current Employment Status**				
Unemployed	1 (2)	0 (0)	1 (7)	0 (0)
Employed	40 (77)	9 (47)	13 (84)	18 (100)
Retired	11 (21)	10 (53)	1 (7)	0 (0)
**Professional Experience**,** (years)**	-	-	-	6 (4–30)

*All values are expressed as frequency (percentage) except for age and professional experience which are expressed as median (range)

Six focus groups sessions were held, two with patients, two with caregivers, and two with healthcare providers. The focus groups were conducted in-person at the two study sites in a location that allowed for privacy and maintenance of confidentiality. A semi structured discussion guide was developed. It focused on three domains of influence from the socio-ecological model (individual, interpersonal, and organizational) [6], to elucidate participants’ experiences and opinions related to barriers and facilitators of prostate cancer care. Sample questions of the discussion guide are presented on Table 2.

**Table 2:** Sample questions on the focus group discussion guide

**DETECTION**
**Individual level** 1. Please describe, from your experience, the current state of localized prostate cancer management in Nigeria? 2. What are your thoughts or beliefs about the causes of prostate cancer?
**Interpersonal level** 1. How would you describe your interaction with caregivers? 2. Can you please describe the efficiency of current mode of communication between referring doctor and specialists?
**Organizational level** 1. What are your views of the way prostate cancer screening should be done in Nigeria (and why)? 2. What are your thoughts about the quality of care that you received for prostate cancer diagnosis? 3. What are your thoughts on the quality and timing of oncological referrals you get from primary, secondary and tertiary levels of care? 4. How has your prostate cancer diagnosis impacted your financial situation or that of your family?
**MANAGEMENT**
**Individual level** 1. How did you make the final choice about treatment to pursue for prostate cancer? 2. How do you feel about the treatment for prostate cancer that you pursued? 3. What were your greatest emotional concerns since the diagnosis of prostate cancer and why were these feelings important? 4. How are treatment guidelines incorporated into regular clinical practice in your institution?”
**Interpersonal level** 1. How would you describe your interaction with other patients with prostate cancer or their caregivers? 2. Can you please share your experience of communication with your prostate cancer doctor? 3. Can you please describe the efficiency of current mode of communication between specialists in different disciplines?
**Organizational level** Finally, thinking about our healthcare system for taking care of prostate cancer patients, what went well with your care? What problems have you faced? How can this be improved? 1. How would you describe the process of clinic appointments and consultation in terms of speed and co-ordination? 2. How do you think that the cost and insurance coverage of investigation and treatment affects the health seeking behaviour of patients with prostate cancer? 3. Describe the system for follow up for prostate cancer patients in your institution?

The moderator for the sessions, one at each site, were researchers with clinical experience, trained in the facilitation of FGDs, communicates fluently in the common language of the site, and understands the relevant clinical and environmental context of the area. The moderator welcomed the views and engagement of each participant and took time to repeat what was said in the local language used by participants to ensure that the points that the participants’ expressed were correctly noted. The discussions were audio recorded, transcribed, and then de-identified to maintain anonymity. Saturation was achieved during the sessions. The mean duration of the focus group discussions was 125 min (range, 110–141 min).

### Data analysis

Qualitative analysis, using a hybrid deductive and inductive approach, was done using NVivo version 12 (QSR International Pty Ltd., Burlington, Massachusetts) based on the framework method of thematic analysis [8]. A codebook was developed based on the socioecological model, other a priori concepts in the discussion guide, and emergent themes that arose during the initial open coding of the transcripts. Based on this codebook, two investigators with different areas of expertise individually coded each transcript. Intercoder reliability was high, with an average agreement of 98.7%. Themes were mapped onto the individual, interpersonal, and organizational levels of the overarching socioecological model. The trustworthiness strategy of this study involved the iterative discussion of findings and their meanings among the research team during fortnightly meeting sessions.

## Results

**Fig. 1:**
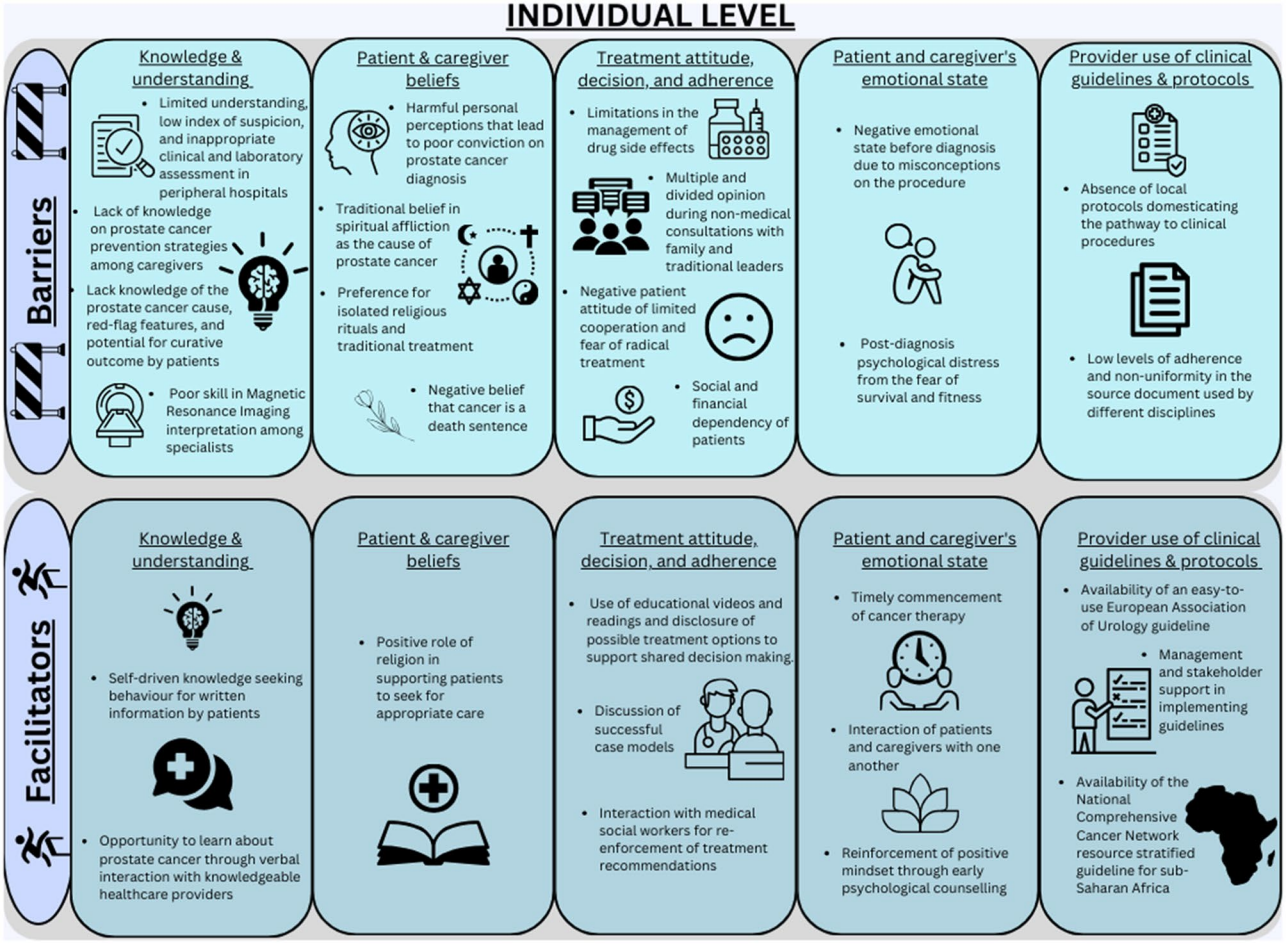
Barriers and facilitators to localized prostate cancer detection and management at the individual level

**Fig. 2:**
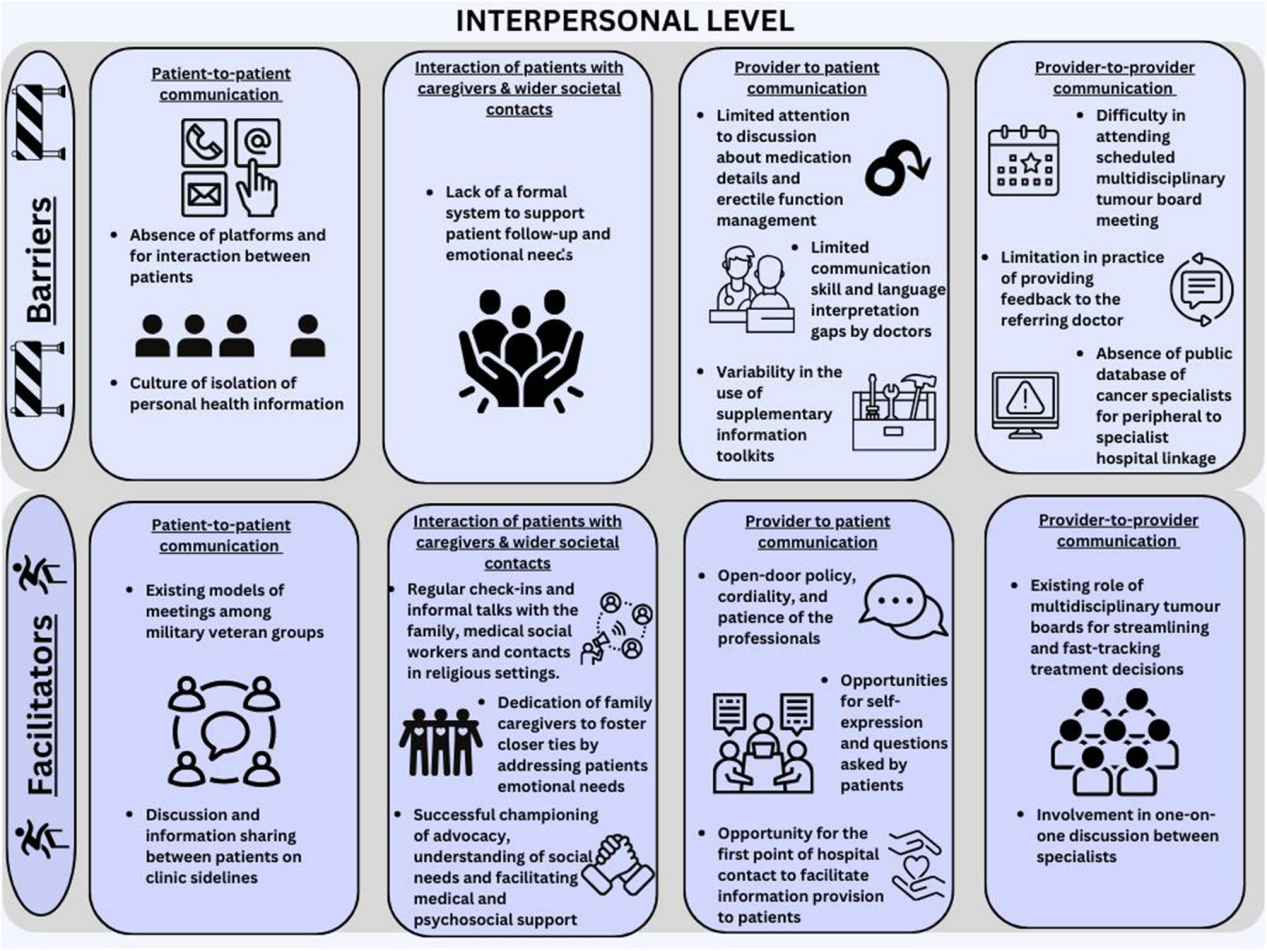
Barriers and facilitators to localized prostate cancer detection and management at the interpersonal level

**Fig. 3:**
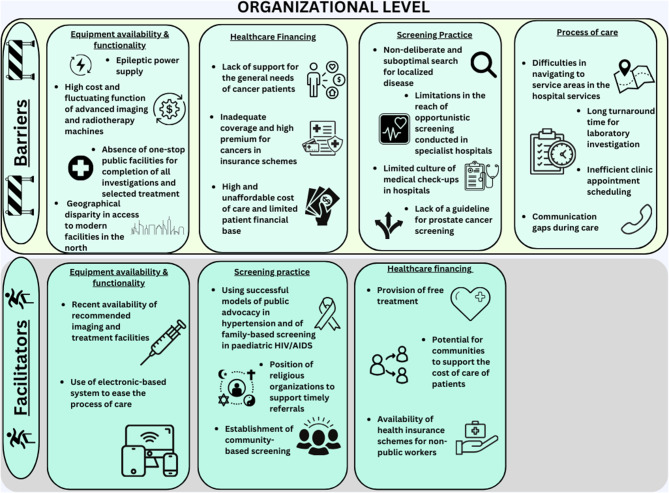
Barriers and facilitators to localized prostate cancer detection and management at the organizational level

Overall, participants spoke more about factors within the individual and interpersonal levels of the socioecological framework compared to organizational level factors. They identified similar numbers of barriers and facilitators, which were often related and led to recommended solutions. Summaries of the main themes are presented on Figs. 1, 2 and 3.

## Detection of prostate cancer

### Individual level

#### Knowledge and understanding of prostate cancer

Knowledge gaps in prostate cancer detection were mentioned more frequently among patients. One of the patients (male, 65 years) declared: *“I did not know the symptoms and signs to look out for in prostate cancer”*. Family caregivers, however, focused more on their lack of knowledge about Prostate Specific Antigen screening as a preventive strategy. Patients, caregivers, and providers cited instances of misinformation, low index of suspicion, and inappropriate clinical tests as barriers among untrained health practitioners in the community setting. A urology specialist (male, 52 years) stated: *“When they go to other medical personnel instead of trained specialists for the disease*,* proper assessment is not made to identify prostate cancer”*. One of the patients (male, 66 years) suggested awareness and advocacy to address this problem noting that: *“There is a lot of awareness about diabetes and hypertension. Doctors should try to make more noise about PSA screening too”*.

#### Beliefs about prostate cancer

Widespread negative beliefs about the cause of prostate cancer, such as spiritual afflictions, were commonly discussed among the patient and caregiver groups in Nigeria, contributing to the non-acceptance of diagnosis. Participants in all categories described negative perceptions of the disease that led to denial or apathy towards diagnosis. For example, a patient (male, 72 years) asked: *“Why should you say that I have prostate cancer when I am asymptomatic”*, and a urology specialist (male, 38 years) made reference to the widely circulated notion that *“cancer is a death sentence”*. However, participants in all stakeholder categories noted that religious tenets also admonish seeking appropriate care.

### Interpersonal level

#### Interaction of patients with caregivers and wider societal contacts

All groups articulated the positive yield of communication between patients and family and medically informed societal contacts in religious settings. For example, a family caregiver (female, 30 years) noted: *“I was also talking to him to be his comfort and everything”*. Caregivers placed particular emphasis on their role in the patient’s cancer journey before early detection and referenced benefits of family and community support such as championing pre-screening health education and advocacy. Another family caregiver, an elderly educationist (female, 75 years), narrated: *“We tell any man above 40 years that comes to our house to go and check their prostate”*.

##### Provider-to-provider communication for upward referrals

Patients indicated that there was no public database of prostate cancer specialists to facilitate the connection of peripheral healthcare providers to the specialists. One of the patients (male, 72 years) stated: *“The data on the professionals that are here is not widespread enough for non-specialist doctors to easily check to know about which doctors are available for the management of a disease”*. A urology specialist (male, 45 years), therefore, advocated for the prioritization of follow-up during the process of early diagnosis in order to address this problem. Another provider, an oncology specialist (male, 50 years) further suggested, *“Our religious organizations have a lot of roles to play in supporting referral of those with certain complaints or those that are diagnosed to the appropriate doctor”*.

### Organizational level

#### Screening practice

Healthcare providers explained that the curative management of prostate cancer was hampered by non-deliberate and suboptimal screening for localized disease. A pathology specialist (male, 36 years) cited the incidental nature of most localized diagnoses while a urology specialist (male, 45 years) highlighted the limitations in the reach of opportunistic screening presently conducted by specialists as *“we cannot get the number that we need to improve early prostate cancer diagnosis especially because majority of patients do not present with symptoms”*. Another urology specialist (male, 42 years) explained that this barrier results from the limited culture of routine medical check-ups for health maintenance and suggested that to close this gap, *“Community based screening is where we need to look towards”*. All stakeholder categories further pointed out that low health insurance coverage and poor financial resource of patients was a barrier to screening and diagnosis. As a consequence of this challenge, an oncology nurse (female, 30 years) noted that: *“People will not want to come to the hospital”*.

A summary of the suggestions for improvement in prostate cancer detection is presented on Table 3.

**Table 3:** Themes and recommended solutions for prostate cancer detection

Levels and Themes	Recommended Solutions
**Individual level**	
Knowledge and understanding of prostate cancer	Early diagnosis awareness through health talks and leaflets distribution in religious houses, schools and rural communities
	Training of the community-level healthcare workforce on warning signs and referral indications
**Interpersonal level**	
Provider-to-provider communication	Creation of referral linkages between doctors in the peripheral and tertiary hospitals
**Organizational Level**	
Screening practice	Creating community-based screening programs
	Increased advocacy for prostate cancer to a level comparable with diabetes and hypertension
	Introduction of community-based cancer information desks and investigations within the skillset of the community-based healthcare workers
	Extending the successful model of family-based screening used in HIV paediatric population to middle-aged relatives of prostate cancer patients.
	Involving religious organizations to support timely referrals
Healthcare financing	Provision of free PSA tests and medical check-ups
	Philanthropy
	Crowdfunding / Community-based cost-sharing

## Management of prostate cancer

### Individual level

#### Treatment attitude, decisions, and adherence

Discussion about facilitators of treatment adherence was predominant in all groups. A medical social worker (male, 32 years) cited an example of his re-enforcement of treatment recommendations through interaction with a patient, while an oncology specialist (male, 50 years) suggested regular follow-up reminders to strengthen patients’ treatment adherence.

Nevertheless, participants also reported negative attitudes toward treatment. Patients (males, 74 and 76 years) cited concerns about radical treatment in the young and surgical fitness in the older adults while an oncology specialist (female, 40 years) emphasized the aversion of patients *“when they hear of the risk of erectile dysfunction”* as a barrier to optimal treatment decisions and adherence. Other patients (males, 70 and 74 years) noted that being scared was a detriment to optimal treatment while caregivers (females, 57 and 65 years) further described uncooperative attitudes among patients. This is sometimes because “*they are in denial of the diagnosis*” (urology specialist, male, 42 years).

#### Emotional state

Overall, there were fewer discussions related to the emotional impact of prostate cancer. One patient (male, 56 years) noted that following diagnosis, *“I felt that everything in my life was gone”*, whereas other patients expressed positive emotions. Many caregivers expressed, in strong emotional tones, a feeling of confusion and sadness due to the psychologically overwhelming burden. For example, one of the family caregivers (female, 30 years) said: “It was not easy emotionally because my mum is late. I cried so much in the bathroom”. Patients and caregivers reflected that these negative states could adversely affect the motivation of patients to seek health care. A patient (male, 56 years) however noted that psychological counselling supported the strengthening of resilience in surmounting the distress when he was “losing control of everything”.

#### Provider use of clinical guidelines and protocols

Healthcare providers acknowledged the availability of international resource-stratified consensus guidelines for prostate cancer management but described practical examples pointing to low levels of adherence to guidelines and non-uniformity in the source document used. A urology specialist (male, 42 years) cited that “*most urologists in Nigeria tend to use the European Association of Urology guidelines*” in contrast to an oncology specialist (male, 40 years) who noted that “*If there are any guideline that I really use as an oncologist*,* it is the National Comprehensive Cancer Network guideline*”. This led to a significant variation in treatment recommendations among providers (oncology specialist, male, 63 years). The oncology specialist (male, 40 years) therefore stressed the need for institutional support to strengthen guideline adherence. A pathology specialist (male, 36 years) further highlighted the need for more locally generated evidence by “*communicating with the department and people involved in order to identify and consider available resource and personnel peculiarities*”.

### Interpersonal level

#### Patient-to-patient communication

Participants placed greater emphasis on barriers to patient-to-patient communication than facilitators. Patients unanimously highlighted the absence of platforms to support interaction among patients. One patient (male, 68 years) noted: *“Everybody is just on their own”*. However, patients referenced models, such as meetings among a military veteran group (male, 70 years) and face-to-face discussion on clinic sidelines (males, 56 and 74 years), as successful facilitators of peer interaction. A urology specialist (male, 42 years) therefore advocated for the creation of a formal patient support group “*where patients can exchange experiences and see that what the doctor is saying is actually true*”.

#### Interaction of patients with caregivers and medical social workers

All groups articulated the positive yield of communication between patients and family caregivers or medical social workers. Caregivers discussed their positive role in providing more context about the patient during clinic consultation and in the delivery of psychosocial support. For example, one of them (female, 66 years) said: *“I told him that I’ll go through it with him*,* and we are going to see it to the end together. That assurance created some relief for him”.* A patient (male, 69 years) also referenced benefits such as getting timely medication reminders from caregivers.

#### Provider-to-patient communication

Provider-to-patient communication was the most dominant facilitator discussed across the three stakeholder categories. Patients described the value of face-to-face discussions (male 74 years), short messaging service (male, 62 years), and printed documents (males, 56 and 69 years). Caregivers (females, 66 and 65 years) cited the importance of phone calls as channels of communication between providers and patients. An oncology provider (female, 40 years) highlighted the advantage of this information exchange noting that: *“you can actually tell them that their case is curable. It makes them relaxed and happy”*. However, other providers such as the urology specialist (male, 42 years) noted the hurdle of lack of interpreters while the pathology specialist (male, 60 years) reflected that providers have communication skill deficits and suggested implementing communication training for providers. Patients further made reference to time limitations during deep conversations with doctors, variable access to education materials, and difficulty in obtaining timely response to information needs. One of the patients (male, 56 years) identified opportunities for ancillary staff, such as nurses to facilitate information provision. A medical social worker (male, 32 years) reiterated: “*Many patients come to seek advice. I usually go back to the managing clinician to get further clarity on the patient. I then advice and counsel the patients*”.

#### Provider-to-provider communication in multidisciplinary teams

Healthcare providers emphasized that tumour boards were the fulcrum of provider-to-provider communication. A urology specialist (male, 52 years) explained that tumour boards provided an opportunity to brainstorm on the best clinical management option for patients and fast-track the decision-making process while stating that it was difficult to achieve complete attendance due to the conflicting activities of providers. An oncology specialist (male, 33 years) further indicated that the communication was not optimal because of the tight schedule of providers.

### Organizational level

#### Process of care

Patients and caregivers strongly articulated several shortcomings during the process of their care citing examples of inefficient clinic appointment systems characterized by long waiting times, delays in the release of test results, and the significant difficulty and loss of energy experienced by sick patients while navigating the vast hospital complex to retrieve hospital cards, make payments, and submit blood and tissue samples. As a result of the long waiting times, a caregiver (female, 57 years) explained, *“Many people don’t want to come to the hospitals*,* especially these big ones”*. Patients and caregivers further identified public transportation through relatively long distances to the hospital as another disincentive to achieving optimal care. A patient (male, 56 years) therefore advocated for the presence of navigators who will direct and support patients during the transition between points of care.

#### Healthcare financing of treatment

All stakeholder categories also discussed the significant limitation posed by the low health insurance coverage and financial resources of patients to treatment services. One of the patients (male, 66 years) attributed this to their vulnerable status as senior citizens on little or no pension schemes, while an oncology nurse (female, 30 years) ascribed it to the exclusion of most oncology-directed treatment in the popular public health insurance scheme.

#### Other themes at the organizational level

Patients and providers emphasized several deficits in “equipment availability and function” including geographical disparity in access to cancer care facilities in northern Nigeria that pose serious challenges to timely care. Patients (males, 59 and 66 years) also saliently noted the limited number of oncologists. An oncology specialist (male, 63 years) also highlighted the limited number of psychologists who were considered important in helping patients cope with *“a tumour that can affect patients’ sexual activity”*. A urology specialist (male, 38 years) thus suggested the introduction of basic psychological screening by oncologists to “give hope to the patients”. Finally, the need for “Data registry and clinicopathological documentation” also emerged as a theme at this level.

A summary of suggestions for improvement in prostate cancer management is presented on Table 4.

**Table 4:** Themes and recommended solutions for prostate cancer management

Levels and Themes	Recommended Solutions
**Individual level**	
Treatment attitude, decision, and adherence	Re-enforcement of treatment recommendation through interaction with medical social worker
	Regular follow-up reminders
Emotional state	Psychological screening and counselling in the early phase at diagnosis and during the treatment and survivourship period
	Timely commencement of cancer therapy
	Interaction of patients and caregivers with one another
Provider use of clinical guidelines and protocols	Domestication of the evidence based on patient needs, affordability, and resource availability.
	Need for institutional support
**Interpersonal level**	
Patient-to-patient communication	Establishment of a formal patient support group.
Provider-to-patient communication	Information provision by the first patient point of contact, such as nurses and medical social workers
	Healthcare provider communication training
Provider-to-provider communication	Development of management protocols
	Selective discussion of complex cases
	Adoption of real-time tumour board discussion in a dedicated WhatsApp group.
**Organizational Level**	
Process of care	Presence of individuals who will direct and support patients during the transition between points of care.
Healthcare financing of treatment	Expanded enrolment in voluntary insurance schemes at the point of contact
	Provision of free drugs
Human resource capacity	Basic psychological screening by oncologists and the selective referral of complex cases to psychologists
Data registry and clinicopathological documentation	Use of patient information sheets for documentation

## Discussion

Improvement in the pathways to detection and management is vital to improvement in survival outcomes of cancers [9]. Interventions designed to improve these pathways should be based on stakeholder input. This study therefore explored the unique viewpoints of patients, caregivers, and providers on the determinants of successful detection and management of clinically localized prostate cancer at two designated comprehensive cancer centres in Nigeria.

Regarding prostate cancer detection, the finding that determinants at the individual level of the socioecological model were more dominant reflects a major need for improvement in patients’ awareness of prostate cancer and in their help-seeking behaviour. Gaps in knowledge and understanding and the negative beliefs of patients and caregivers about prostate cancer suggest that there is a low level of prostate cancer health literacy among patients and caregivers. Similar to this study, Kaninjing at al. and Ezenwankwo et al. have reported poor awareness of prostate cancer and its red-flag symptoms and disease misattribution due to reliance on folklore and myths as barriers to care-seeking in the Nigerian context [10, 11]. This study further linked knowledge barriers among healthcare providers at the primary and secondary levels of care to timely diagnosis, therefore, providing a potentially actionable target for improvement in prostate cancer early detection in Nigeria.

Another challenge of prostate cancer early detection highlighted in this study is the relative centralization in the provision of cancer assessment at tertiary levels of care in Nigeria. Community stakeholders, in the study of Adedeji et al. [12], noted that the absence of prostate cancer screening centres, across the local government areas that serve rural dwellers, was a barrier to the access of this preventive care service. The perspective of providers in this study, therefore, reinforces the opinion of community stakeholders in their study. Moreover, this study further describes the difficulties in the referral of patients from community-level healthcare centres to specialist hospitals. These organizational and interpersonal-level gaps has a negative impact on easy and early access to prostate cancer detection in the communities [13].

Turning to prostate cancer management, this study highlighted the emotional, logistical, and financial difficulties faced by patients during the processes of care. The distress that they encounter during the journey through laboratory tests, imaging investigations, radical prostatectomy, radical radiotherapy, and survivourship can adversely affect patient motivation to continue receiving care. Despite these challenges, the great dominance of interpersonal-level facilitators, such as the role of peer support among patients, family support, nurses, and medical social workers in assisting patients to overcome barriers to care and get the support needed, stood out as a finding in this study. Kim et al. in South Africa also observed the pains faced by patients living with prostate cancer and the role of social and emotional support in fostering coping and resilience [14]. These mechanisms can be leveraged as an asset to improve the quality of patient-centred care during the management of prostate cancer in Nigeria.

At the organizational level of prostate cancer management, limited provider capacity for psychological care represents a salient and important need, especially because it closely relates to the negative emotional state of some individual patients during their post-diagnosis journey. Unlike this study, where distress was related to peri-diagnosis shock and the prospect of erectile dysfunction, Kim et al. [14] identified stigma as a key challenge encountered by prostate cancer patients. It, therefore, appears that the setting of South Africa, where cancer and HIV are regarded as secret conditions, is different from the cultural context of Nigeria. It could also be that the risk of stigma is mitigated by the curative focus for prostate cancer in this present study. Participants in this study also identified other challenges at the organizational level, such as long travel distances, equipment breakdown, and inadequate health insurance programs, which have been documented as general barriers of access to cancer care in other studies in Nigeria [13, 15–17].

The result of this needs assessment is important during the process of intervention development in mapping the contextual barriers and facilitators identified by stakeholders to the potential steps that can be taken to address the problems [18]. The study participants recommended solutions in the areas of prostate cancer early detection, patient navigation, guideline-based management, and basic psychological care. These represents expected changes in behaviour and environment that will be mapped to determinants, and used during brainstorming and prioritization sessions to develop a final list of systems strengthening intervention strategies for localized prostate cancer detection and management in Nigeria.

This study should be interpreted within the lens of some limitations. Because the stakeholders were purposively selected, their views may not be generalizable to the population. In addition, the opinions of community and religious leaders as well as those of policymakers were not included in this study. Nevertheless, the strength of this study lies in the use of a multi-level socio-ecological approach to deeply understand barriers and enhancers of care in a complex setting.

## Conclusions

This study identified multi-level determinants that may affect the optimal diagnosis, treatment, follow-up, survivourship and secondary prevention of localized prostate cancer in Nigeria. Stakeholder priorities in the areas of early detection, patient navigation, guideline-based management and basic psychological care are recommended as targets of future interventions. This study will be used to inform implementation research on the development of these multi-faceted implementation strategies in order to improve the quality of prostate cancer detection and management in Nigeria.

## Data Availability

The datasets used and/or analysed during the current study are available from the corresponding author on reasonable request.
